# Supplementary material for: Active travel to school: a longitudinal millennium cohort study of schooling outcomes
Source: BMJ Open. 2023 Mar 23;13(3):e068388. doi: 10.1136/bmjopen-2022-068388 (PMC10040056; doi:10.1136/bmjopen-2022-068388)
Supplement: Author's manuscript [file bmjopen-2022-068388.draft_revisions.pdf]

BMJ Open

BMJ Open is committed to open peer review. As part of this commitment we make the peer review history of every article we publish publicly available.

When an article is published we post the peer reviewers' comments and the authors' responses online. We also post the versions of the paper that were used during peer review. These are the versions that the peer review comments apply to.

The versions of the paper that follow are the versions that were submitted during the peer review process. They are not the versions of record or the final published versions. They should not be cited or distributed as the published version of this manuscript.

BMJ Open is an open access journal and the full, final, typeset and author-corrected version of record of the manuscript is available on our site with no access controls, subscription charges or pay-per-view fees (http://bmjopen.bmj.com).

If you have any questions on BMJ Open's open peer review process please email info.bmjopen@bmj.com

BMJ Open

Active travel to school: a longitudinal Millennium Cohort study of schooling outcomes

Journal:	*BMJ Open*
Manuscript ID	bmjopen-2022-068388
Article Type:	Original research
Date Submitted by the Author:	16-Sep-2022
Complete List of Authors:	Walker, Ian; University of Surrey, School of Psychology<br>Gamble, Tim; University of Surrey, School of Psychology
Keywords:	PUBLIC HEALTH, PREVENTIVE MEDICINE, SPORTS MEDICINE

SCHOLARONE™
Manuscripts

I, the Submitting Author has the right to grant and does grant on behalf of all authors of the Work (as defined in the below author licence), an exclusive licence and/or a non-exclusive licence for contributions from authors who are: i) UK Crown employees; ii) where BMJ has agreed a CC-BY licence shall apply, and/or iii) in accordance with the terms applicable for US Federal Government officers or employees acting as part of their official duties; on a worldwide, perpetual, irrevocable, royalty-free basis to BMJ Publishing Group Ltd ("BMJ") its licensees and where the relevant Journal is co-owned by BMJ to the co-owners of the Journal, to publish the Work in this journal and any other BMJ products and to exploit all rights, as set out in our licence.

The Submitting Author accepts and understands that any supply made under these terms is made by BMJ to the Submitting Author unless you are acting as an employee on behalf of your employer or a postgraduate student of an affiliated institution which is paying any applicable article publishing charge ("APC") for Open Access articles. Where the Submitting Author wishes to make the Work available on an Open Access basis (and intends to pay the relevant APC), the terms of reuse of such Open Access shall be governed by a Creative Commons licence – details of these licences and which Creative Commons licence will apply to this Work are set out in our licence referred to above.

Other than as permitted in any relevant BMJ Author's Self Archiving Policies, I confirm this Work has not been accepted for publication elsewhere, is not being considered for publication elsewhere and does not duplicate material already published. I confirm all authors consent to publication of this Work and authorise the granting of this licence.

For peer review only

Active travel to school: a longitudinal Millennium Cohort study of schooling outcomes

Ian Walker[1] and Tim Gamble[1]

[1]School of Psychology, University of Surrey, Guildford, Surrey GU2 7XH, UK

Correspondence to Professor Ian Walker; i.walker@surrey.ac.uk

For peer review only

Word count: 1885 words excluding title page, abstract, references, figures and tables

For peer review only - http://bmjopen.bmj.com/site/about/guidelines.xhtml

ABSTRACT

Objectives To assess longitudinal associations between active travel during the school commute and later educational outcomes.

Setting England, Wales and Northern Ireland.

Participants 6778 children, surveyed at ages 7, 11, 14, and 17, in the UK Millennium Cohort Study.

Primary and secondary outcomes School-leaver GCSE exam scores.

Results After controlling a range of sociodemographic and health variables, using active vs passive travel modes during a child's commute to school during earlier years predicted differences in school-leaver exam performance at age 16. These effects were mediated through changes in self-esteem, emotional difficulties and behavioural difficulties. Examples include: being driven to school at 11 was associated with improved exam performance at 16 mediated through enhanced self-esteem at 14 (*ab* = 0.08, 95% CI = 0.00 to 0.18, *p* = .05); public transport at 14 was associated with better exam scores at 16, mediated through enhanced self-esteem at 16 (*ab* = 0.19, 95% CI = 0.05 to 0.37, *p* = .01); cycling at 14 was associated with worse exam scores mediated through greater behavioural problems at 16 (*ab* = -0.78, 95% CI = -1.52 to -0.15, *p* = .03), although cycling at 14 was also separately associated with better exam scores at 16 mediated through reduced emotional difficulty at 16 (*ab* = 0.10, 95% CI = 0.01 to 0.30, *p* = .05). Importantly, although our model predicted 21% variance in exam performance, removing travel mode barely reduced its ability to predict exam scores ($\Delta R^2 = -.005$, $F_{20,6469} = 2.50$, $p < .001$).

For peer review only - http://bmjopen.bmj.com/site/about/guidelines.xhtml

Conclusion There are differences in school-leaver exam performance linked to travel mode choice earlier in the school career, but these differences are extremely small. There appears to be no realistic educational disadvantage from any given travel mode, strengthening the case for cleaner, healthier modes to become the default.

Keywords: Environment and Public Health; Schools; Child Psychology; Travel; Cohort Analysis

For peer review only

For peer review only - http://bmjopen.bmj.com/site/about/guidelines.xhtml

ARTICLE SUMMARY

Strengths and limitations of this study

- Strength: We used the high-quality UK Millennium Cohort dataset, following a large sample of children over 10 years, enabling us to consider a broad range of health and demographic covariates.
- Strength: We used official, national-standard exam results as the outcome measure.
- Limitation: Data on travel to school were self-reported and reduced each child to a single 'main' mode, thereby not fully capturing the details of children whose journeys were more complex.
- Limitation: Data collection was at three-year intervals and so this dataset would not allow us to identify specific ages that are critical in development at anything other than a crude level.

For peer review only

INTRODUCTION

A child's daily journey to school provides routine opportunities for physical activity that might have important health consequences[1–3]. Active travel modes such as walking, cycling and scooting provide valuable daily exercise that is absent when children are driven to school, to say nothing of how driving children to school creates further serious public health problems through air-quality impairment. As well as obvious barriers to the adoption of more active travel for the school commute, such as geography and societal norms, there is a further barrier arising from the informal learning opportunities linked to the daily commute[4]. It has long been claimed that children who travel to school passively – like being chauffeured in cars – can miss out on everyday learning opportunities during their commutes compared to children who travel independently[4–6], and might also be less attentive upon arrival at school than children who use active modes like walking[3,7]. This might appear to make active travel the obvious choice except, in direct contrast to the studies just mentioned, it has also been claimed that being driven to school by adult caregivers every day provides children with important educational opportunities[6,8,9]. These conflicting claims, and a lack of evidence, mean policymakers cannot know the wider effects of supporting sustainable active travel to school (as is legally mandated in countries like the UK[10]). A strong test of this issue would be to ask whether any effects of travel to school can be picked up in official end-of-school exam grades, and this is what we provide here.

Although the mechanisms by which everyday travel might affect learning at school[4–6] have been discussed since the early 1990s[4–6], evidence in this area remains weak[11,12]. Five components of childhood wellbeing have been distinguished (physical, psychological, social, economic and cognitive[13]) – the last of which provides a plausible route between physical activity and school

For peer review only - http://bmjopen.bmj.com/site/about/guidelines.xhtml

performance[11]. A recent comprehensive review[12] suggested that cognitive ability, and thereby academic attainment, might be influenced by children's experiences on the journey to school through mechanisms like increased alertness[3] in walkers, cyclists and bus users, but noted that evidence remains anecdotal or inconsistent, a point also made recently elsewhere[11]. Enhanced peer relationships are another mechanism suggested to explain how independent travel might benefit children who walk or take buses to school[6,12], but there are also qualitative data from multiple sources suggesting that regular parental interaction for children who are escorted in cars could have educational benefits[6,8,9]. The contradictory claims in this area are impossible to resolve given the existing evidence[11,12] and so we performed a direct test of whether how children travel to school could predict their end-of-school exam scores several years later. Given the past research outlined here, we hypothesized that if we saw any educational advantages for users of certain travel modes, these might be separately mediated through changes in behavioural/attentional problems (which might plausibly be improved by regular physical activity[3,7,11,12]) and self-esteem (which might plausibly be improved from experiences of autonomy in school travel[4,6,7,11,12]).

For peer review only

METHODS

We tested our hypotheses using the UK Millennium Cohort dataset[16], which tracks people from their birth in 2000 to adulthood. We had data from 7009 participants: 5187 from England, 1058 from Wales and 764 from Northern Ireland (Scotland's exam system is different). Of these, 6778 provided complete data on GCSE (General Certificate of Secondary Education) or iGCSE (international GCSE) exams at 16, plus their history of school travel. We were able to calculate a

For peer review only - http://bmjopen.bmj.com/site/about/guidelines.xhtml

total numeric school-leaving exam score for each person using a government points system[17] to translate letter grades into numerical equivalents, giving greater credit to those who obtained higher grades and those who chose to take extra exams. The mean exam score for the sample was 46.27 points (SD = 21.87) which roughly corresponds to a child obtaining 5 B grades and 4 C grades[17].

We ran a series of causal mediation analyses[18] to assess plausible mechanistic pathways from travel mode to exam performance. We tested three potential mediators: Externalizing, which is a collective measure of inattentiveness, hyperactivity and conduct problems from the Strengths and Difficulties Questionnaire (SDQ)[19]; SDQ Internalizing, which measures emotional and peer-relationship difficulties[19]; and the Rosenberg Self-Esteem measure[20]. Each mediation analysis controlled for demographics and behavioral factors including sex, ethnicity, country of residence, distance from school, household income quintile, private schooling and – because physical activity was a plausible mechanism behind any travel-mode effects on educational success[3,8–10] – leisure-time sport and incidental physical activity. The following temporal chains were assessed: travel at 7 → mediators at 11 → exams at 16; travel at 7 → mediators at 14 → exams at 16; travel at 11 → mediators at 14 → exams at 16; and travel at 14 → mediators at 16 → exams at 16. Mediation coefficients *ab* are interpreted as the mean marginal (i.e., controlling for covariates) change in exam score associated with the travel mode in question which is explained specifically through changes in the mediating variable[18]. Walking was set as the reference travel mode. Confidence intervals were estimated using nonparametric bootstrapping with 500 repetitions.

Linear regression analysis predicted total exam score for each child from travel behavior at 7, 11 and 14 using the same demographic and behavioral covariates as the mediation analyses. The

For peer review only - http://bmjopen.bmj.com/site/about/guidelines.xhtml

covariates were also tested for moderation effects with transport mode. A process of model simplification used backward deletion to remove redundant predictors and interaction terms and so leave the minimal adequate model.

RESULTS

Our analysis found pathways between school travel mode and later exam performance, separately mediated through self-esteem, externalized behavior problems and internalized socio-emotional difficulties. The pathways with significant indirect mediated effects were as follows: cycling at 7 was associated with lower exam performance at 16 mediated through self-esteem changes at 11 (*ab* = -0.80, 95% CI = -1.68 to -0.07, *p* = .04); being driven at 11 was associated with improved exam performance at 16 mediated through enhanced self-esteem at 14 (*ab* = 0.08, 95% CI = 0.00 to 0.18, *p* = .05); being driven at 14 was associated with better exam performance at 16 mediated through enhanced self-esteem at 16 (*ab* = 0.20, 95% CI = 0.08 to 0.33, *p* < .001); public transport at 14 was associated with better exam scores at 16, also mediated through enhanced self-esteem at 16 (*ab* = 0.19, 95% CI = 0.05 to 0.37, *p* = .01); cycling at 14 was associated with worse exam scores at 16 mediated through greater externalizing at 16 (*ab* = -0.78, 95% CI = -1.52 to -0.15, *p* = .03), although cycling at 14 was also separately associated with better exam scores at 16 mediated through reduced internalizing at 16 (*ab* = 0.10, 95% CI = 0.01 to 0.30, *p* = .05). All other mediated pathways between travel history and exam scores were non-significant (all $p \geq .10$).

Regression analysis (Table 1) found that overall exam performance at 16 was related to the travel mode used to reach school two years earlier, and to demographic variables including sex

For peer review only - http://bmjopen.bmj.com/site/about/guidelines.xhtml

and household income (R^2_{adj} = .21). Many of the control variable effects were expected (e.g., a mean marginal exam advantage of 6.30 points enjoyed by girls[14]), which supports the validity of the analysis. All minority ethnic groups performed substantially better than the reference white category and there were very clear cumulative advantages for children in higher-income households, peaking at a marginal mean difference of 24.46 points for the highest quintile versus the lowest (roughly equivalent to 8 exams each rising from grade C to grade A). The influence of travel mode was significantly moderated by household income quintile (Fig. 1); perhaps the most notable aspect of this interaction was that, in the highest household income group, there was a mean marginal reduction of 4.63 exam points (equivalent to three exams each dropping from an A to a B) for the children who were driven to school.

Critically, however, entirely removing travel mode from our model barely changed its overall ability to predict exam scores (ΔR^2 = -.005, $F_{20,6469}$ = 2.50, p < .001). This reveals travel mode to be a trivial influence on exam scores compared to the demographic and behavioral covariates. This accords with the small effect sizes described above, e.g., being driven at 14 led to a mean mediated change in exam scores of just 0.20 points across all exams versus walking, which is extremely small against an overall mean exam score of 46.27 points.

DISCUSSION

This analysis has shown for the first time that there are specific psycho-behavioural pathways linking travel to school in teenage years with later academic outcomes, as assessed through end-of-school exam results. The pathways were particularly mediated through changes in self-esteem, and to some extent behavioral and socio-emotional difficulties, that in turn were

For peer review only - http://bmjopen.bmj.com/site/about/guidelines.xhtml

influenced by travel experiences earlier in a child's school career. Prior work has distinguished two domains that might be associated with daily travel: psychological (wellbeing, independence) and cognitive (mental ability, academic attainment)[11,12]. The present study has demonstrated that these two domains interact such that the psychological domain might have an effect on the cognitive domain. It is notable that being driven to school by adult caregivers at 14 and independently using public transport at 14 both produced similar effects on later exam performance through a route involving enhanced self-esteem. This implies that, contrary to past claims[4–6,8,9], it cannot simply be quality time with adults or independent mobility that uniquely boosts school success. Rather, there appears to be more than one route to better school outcomes as a result of daily travel experiences and both active and passive modes can provide some of these benefits provided they have immediate effects on mediating psychological factors like self-esteem, emotional adjustment or behavioral difficulties.

That last point notwithstanding, this study's core finding is to show, using an adequately powered sample, that while components of childhood wellbeing like self-esteem and socio-emotional difficulties[11–13] can be influenced by the way a child travels to school, and that while these in turn might influence exam success, the magnitude of this effect on school outcomes is dramatically smaller than the influences of demographics, health and socio-economic status (cf.[12]). Once these covariates were controlled, the way children travelled to school explained just 0.5% of unique variance in exam outcomes. Given the size and quality of the sample used here, we can state with some confidence that the way children travel to school is a real, but very minor, influence on their scholastic success.

For peer review only - http://bmjopen.bmj.com/site/about/guidelines.xhtml

CONCLUSION

Our finding that there is no clear advantage for any one travel mode should end some long-standing speculation within the research literature about the impact of travel experiences on learning[3–9,11–13] and thereby help unlock public health gains by removing an impediment to reducing the use of private cars for the school commute. Modes like walking, cycling and buses are immediately positive for children's wellbeing[15] and also provide health benefits[1–2], so it is perhaps surprising they do not translate into more substantial academic performance advantages. On the other hand, as we have clearly shown here, nor does spending time in cars with adult caregivers. Given the known negative consequences of driving children to school, including air pollution and lack of physical activity[1,2,11], it is now clear that policymakers can recommend active and public transport modes as the default choice without fear of meaningful long-term educational disadvantage.

Acknowledgments We thank Anthony Laverty for his advice on working with the Millennium Cohort dataset.

Author Contributions Conceptualization: IW, TG; Methodology: IW, TG; Data management: TG; Statistical analysis: IW; Visualization: IW; Writing – original draft: IW; Writing – review & editing: IW, TG

Funding This study/project did not receive external funding.

Disclaimer The views expressed are those of the author(s) and not necessarily those of the University of Surrey.

For peer review only - http://bmjopen.bmj.com/site/about/guidelines.xhtml

Competing interests None declared.

Patient consent for publication Not required.

Ethics approval As this study involved secondary analyses of data that do not contain identifiable information, the MCS ethical approval was not required. Data collection for the MCS has ethical approval from the Yorkshire and Humber ethics committee (11/YH/0203) and further details are available from http://www.cls.ioe. ac.uk/. Participants give consent for their data to be used for research purposes.

Provenance and peer review Not commissioned; externally peer reviewed.

Data availability statement MCS data are available from https://ukdataservice.ac.uk/. Study pre-registration available from https://osf.io/u9wdr. Analysis code available from https://osf.io/46eq3.

Supplemental material This content has been supplied by the author(s). It has not been vetted by BMJ Publishing Group Limited (BMJ) and may not have been peer-reviewed. Any opinions or recommendations discussed are solely those of the author(s) and are not endorsed by BMJ. BMJ disclaims all liability and responsibility arising from any reliance placed on the content. Where the content includes any translated material, BMJ does not warrant the accuracy and reliability of the translations (including but not limited to local regulations, clinical guidelines, terminology, drug names and drug dosages), and is not responsible for any error and/or omissions arising from translation and adaptation or otherwise.

Open access This is an open access article distributed in accordance with the Creative Commons Attribution 4.0 Unported (CC BY 4.0) license, which permits others to copy, redistribute, remix,

For peer review only - http://bmjopen.bmj.com/site/about/guidelines.xhtml

transform and build upon this work for any purpose, provided the original work is properly cited, a link to the licence is given, and indication of whether changes were made. See: https://creativecommons.org/ licenses/by/4.0/.

ORCID iDs

Ian Walker https://orcid.org/0000-0002-0079-3149

Tim Gamble https://orcid.org/0000-0002-6610-0835

REFERENCES

1 Tudor-Locke C, Ainsworth BE, Popkin BM. Active commuting to school: an overlooked source of children's physical activity? *Sports Med* 2001;31:309–313.

2 Laverty AA, Hone T, Goodman A, Kelly Y, Millett C. Associations of active travel with adiposity among children and socioeconomic differentials: a longitudinal study. *BMJ Open* 2021;11:e036041.

3 Mackett RL, Lucas L, Paskins J, Turbin J. The therapeutic value of children's everyday travel. *Trans Res Part A* 2005;39:205–219.

4 Kegerreis S. Independent mobility and children's mental and emotional development. In Hillman M, ed. Children, Transport and the Quality of Life. London: *Policy Studies Institute*, 1993:28–24.

5 Hillman M, Adams J, Whitelegg J. One False Move: A Study of Children's Independent Mobility. London: *Policy Studies Institute*, 1990.

For peer review only - http://bmjopen.bmj.com/site/about/guidelines.xhtml

6 Gough D, Oliver D, Brunton G, Selai C, Schaumberg H. The effect of travel modes on children's mental health, cognitive and social development: A systematic review. London: *Evidence for Policy and Practice Information and Co-ordinating Centre*, 2001.

7 Cooper AR, Andersen LB, Wedderkopp N, Page AS, Froberg K. Physical activity levels of children who walk, cycle or are driven to school. *Am J Prev Med* 2005;29:179–184.

8 Barker J. 'Manic mums' and 'distant dads'? Gendered geographies of care and the journey to school. *Health & Place* 2011;17:413–421.

9 Yeung R, Nguyen-Hoang P. It's the journey, not the destination: The effect of school travel mode on student achievement. *J Urbanism: Int Res on Placemaking and Urban Sustainability* 2020;13:170–186.

10 UK Government. Section 508A, Education Act, 1996. Available: https://www.legislation.gov.uk/ukpga/1996/56/section/508A

11 Westman J, Friman M, Olsson LE. Travel and child wellbeing: The psychological and cognitive domains. In Owen E, Waygood D, Friman M, Olsson LE, Mitra R, eds. Transport and Children's Wellbeing. *Elsevier*, 2020:41–59.

12 Waygood EOD, Friman M, Olsson LE, Taniguchi A. Transport and child well-being: An integrative review. *Trav Beh and Soc* 2017;9:32–49.

13 Pollard E, Lee P. Child well-being: A systematic review of the literature. *Social Indicators Research* 2003;61:59–78.

14 Angelo C, Reis AB. Gender gaps in different grading systems. *Edu Econ* 2021;29:105–119.

For peer review only

15 Westman J, Olsson LE, Gärling T, Friman M. Children's travel to school: satisfaction, current mood, and cognitive performance. *Transportation* 2017;44:1365–1382.

16 Connelly R, Platt L. Cohort profile: UK millennium cohort study (MCS). *Int J Epidemiol* 2014;43:1719–1725.

17 UK Department for Education. GCSE factsheet for parents, 2019. Available: https://www.gov.uk/government/publications/gcse-new-grading-scale-factsheets

18 Hayes AF, Preacher KJ. Statistical mediation analysis with a multicategorical independent variable. *Brit J Math Stat Psych* 2014;67:451–470.

19 Goodman R. The strengths and difficulties questionnaire: A research note. *J Child Psych Psychiat* 1997;38:581–586.

20 Rosenberg M. Rosenberg Self-Esteem Scale. *APA PsycTests*, 1965.

For peer review only - http://bmjopen.bmj.com/site/about/guidelines.xhtml

FIGURES AND TABLES

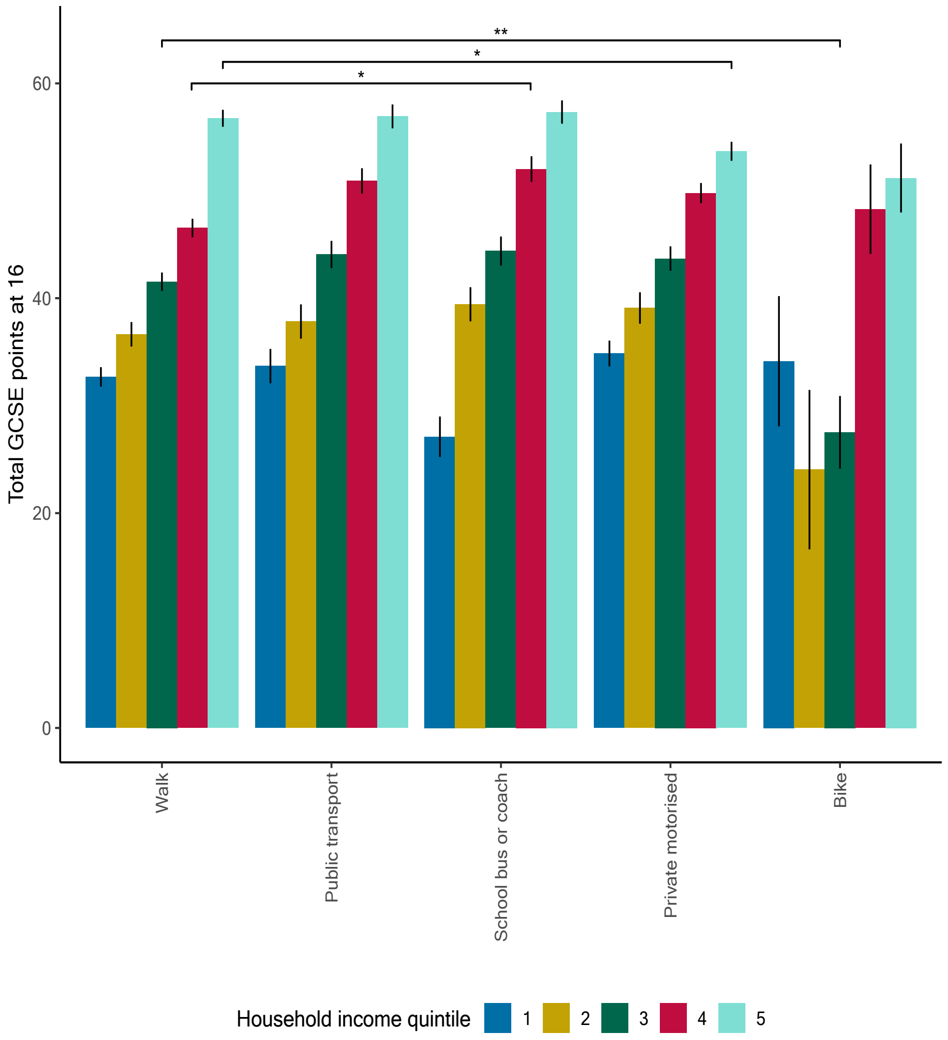

Fig. 1 School exam scores at age 16 from N = 6778 children according to household income quintile and travel mode at age 14. Walking was set as the travel reference category. * $p < .05$, ** $p < .01$

For peer review only - http://bmjopen.bmj.com/site/about/guidelines.xhtml

Table 1. Linear regression model predicting total school exam scores at age 16 from demographic and travel variables

		Coeff.	Std err.	*t*	*p*	95% CI lower	95% CI upper
	(Intercept)	26.48	1.39	19.04	<.001	23.75	29.21
Country	England	ref					
	Wales	4.25	0.72	5.92	<.001	2.84	5.66
	Northern Ireland	3.71	0.84	4.41	<.001	2.06	5.36
Sex	Male	ref					
	Female	6.30	0.49	12.80	<.001	5.34	7.27
Ethnicity	White	ref					
	Mixed	3.44	1.46	2.35	.02	0.57	6.31
	Indian	8.93	1.41	6.32	<.001	6.16	11.71
	Pakistani/Bangladeshi	9.49	1.16	8.17	<.001	7.21	11.76
	Black	4.78	1.51	3.16	.002	1.82	7.75
	Other inc. Chinese	16.37	1.92	8.54	<.001	12.61	20.12
Household income quintile	1 (lowest)	ref					
	2	5.09	1.51	3.36	.001	2.12	8.06
	3	10.28	1.40	7.37	<.001	7.55	13.02

For peer review only - http://bmjopen.bmj.com/site/about/guidelines.xhtml

	4	15.00	1.37	10.97	<.001	12.32	17.68
	5 (highest)	24.46	1.38	17.73	<.001	21.58	27.17
Mental health issue	No	ref					
	Yes	-4.39	1.10	3.99	<.001	-6.55	-2.23
Longstanding illness	No	ref					
	Yes	-2.83	0.73	3.88	<.001	-4.25	-1.40
Private schooling	No	ref					
	Yes	2.70	0.92	2.92	.003	0.89	4.52
Sport at age 7	per 1 point increment	1.60	0.21	7.68	<.001	1.19	2.01
Sport at age 11	per 1 point increment	0.99	0.17	5.67	<.001	0.65	1.33
Other physical activity at 7	per 1 point increment	-0.40	0.15	2.67	.007	-0.70	-0.11
Other physical activity at 11	per 1 point increment	-0.51	0.14	3.52	<.001	-0.80	-0.22
Transport mode age 14	Walking	ref					
	Public transport	2.04	1.92	1.06	.29	-1.73	5.81
	School bus or coach	-2.55	2.57	0.99	.32	-7.60	2.50
	Private motorised	0.18	1.67	0.11	.92	-3.10	3.45
	Cycling	9.11	6.24	1.46	.14	-3.13	21.34

For peer review only - http://bmjopen.bmj.com/site/about/guidelines.xhtml

Age 14 public transport (v. walking) x household income quintile 2 (v. 1)	-1.22	2.73	0.45	.66	-6.57	4.14
Age 14 public transport (v. walking) x household income quintile 3 (v. 1)	-0.91	2.47	0.37	.71	-5.75	3.93
Age 14 public transport (v. walking) x household income quintile 4 (v. 1)	1.13	2.41	0.47	.64	-3.59	5.84
Age 14 public transport (v. walking) x household income quintile 5 (v. 1)	-2.86	2.34	1.23	.22	-7.45	1.72
Age 14 school bus (v. walking) x household income quintile 2 (v. 1)	5.95	3.21	1.86	.06	-0.34	12.25
Age 14 school bus (v. walking) x household income quintile 3 (v. 1)	4.72	2.99	1.58	.11	-1.13	10.58
Age 14 school bus (v. walking) x household income quintile 4 (v. 1)	6.51	2.91	2.24	.03	0.82	12.21
Age 14 school bus (v. walking) x household income quintile 5 (v. 1)	2.00	2.87	0.70	.49	-3.62	7.63
Age 14 private motorised (v. walking) x household income quintile 2 (v. 1)	1.53	2.42	0.63	.53	-3.22	6.27
Age 14 private motorised (v. walking) x household income quintile 3 (v. 1)	0.44	2.17	0.20	.84	-3.81	4.69
Age 14 private motorised (v. walking) x household income quintile 4 (v. 1)	1.62	2.07	0.78	.44	-2.45	5.68

For peer review only - http://bmjopen.bmj.com/site/about/guidelines.xhtml

Age 14 private motorised (v. walking) x household income quintile 5 (v. 1)	-4.63	2.03	2.28	.02	-8.61	-0.65
Age 14 cycling (v. walking) x household income quintile 2 (v. 1)	-16.34	8.45	1.93	.05	-32.90	0.23
Age 14 cycling (v. walking) x household income quintile 3 (v. 1)	-19.63	7.49	2.62	.008	-34.31	-4.95
Age 14 cycling (v. walking) x household income quintile 4 (v. 1)	-2.22	7.25	0.31	.76	-16.43	11.98
Age 14 cycling (v. walking) x household income quintile 5 (v. 1)	-12.85	6.91	1.86	.06	-26.39	0.69

Note: Redundant predictors and interaction terms have been removed.

For peer review only

For peer review only - http://bmjopen.bmj.com/site/about/guidelines.xhtml

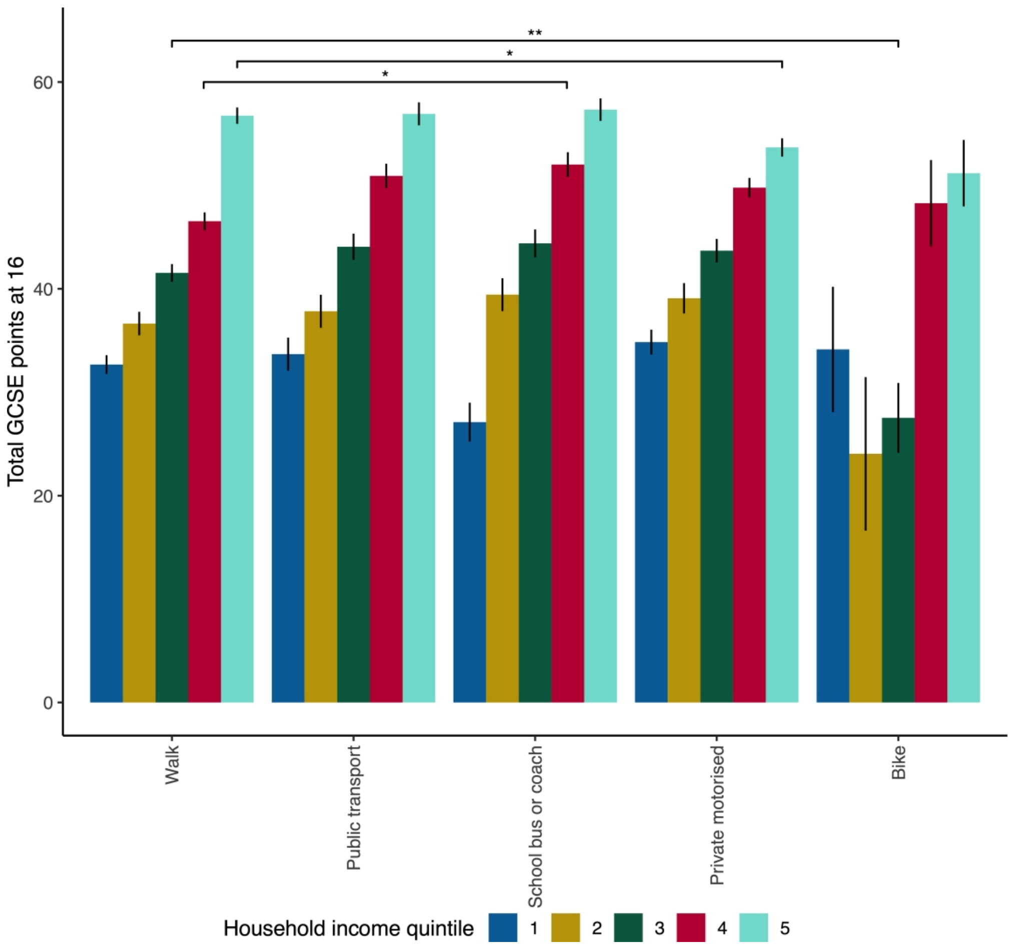

Fig. 1 School exam scores at age 16 from N = 6778 children according to household income quintile and travel mode at age 14. Walking was set as the travel reference category. * p < .05, ** p < .01

1383x1291mm (118 x 118 DPI)

BMJ Open

Active travel to school: a longitudinal Millennium Cohort study of schooling outcomes

Journal:	*BMJ Open*
Manuscript ID	bmjopen-2022-068388.R1
Article Type:	Original research
Date Submitted by the Author:	16-Feb-2023
Complete List of Authors:	Walker, Ian; Swansea University, School of Psychology<br>Gamble, Tim; University of Surrey, School of Psychology
**Primary Subject Heading**:	Public health
Secondary Subject Heading:	Health policy, Sociology
Keywords:	PUBLIC HEALTH, PREVENTIVE MEDICINE, SPORTS MEDICINE

SCHOLARONE™
Manuscripts

I, the Submitting Author has the right to grant and does grant on behalf of all authors of the Work (as defined in the below author licence), an exclusive licence and/or a non-exclusive licence for contributions from authors who are: i) UK Crown employees; ii) where BMJ has agreed a CC-BY licence shall apply, and/or iii) in accordance with the terms applicable for US Federal Government officers or employees acting as part of their official duties; on a worldwide, perpetual, irrevocable, royalty-free basis to BMJ Publishing Group Ltd ("BMJ") its licensees and where the relevant Journal is co-owned by BMJ to the co-owners of the Journal, to publish the Work in this journal and any other BMJ products and to exploit all rights, as set out in our licence.

The Submitting Author accepts and understands that any supply made under these terms is made by BMJ to the Submitting Author unless you are acting as an employee on behalf of your employer or a postgraduate student of an affiliated institution which is paying any applicable article publishing charge ("APC") for Open Access articles. Where the Submitting Author wishes to make the Work available on an Open Access basis (and intends to pay the relevant APC), the terms of reuse of such Open Access shall be governed by a Creative Commons licence – details of these licences and which Creative Commons licence will apply to this Work are set out in our licence referred to above.

Other than as permitted in any relevant BMJ Author's Self Archiving Policies, I confirm this Work has not been accepted for publication elsewhere, is not being considered for publication elsewhere and does not duplicate material already published. I confirm all authors consent to publication of this Work and authorise the granting of this licence.

For peer review only

Active travel to school: A longitudinal Millennium Cohort study of schooling outcomes

Ian Walker[1] and Tim Gamble[2]

[1]School of Psychology, Swansea University, Swansea SA2 8PP, UK

[2]School of Psychology, University of Surrey, Guildford, Surrey GU2 7XH, UK

Correspondence to Professor Ian Walker: ian.walker@swansea.ac.uk

Word count: **2407** words excluding title page, abstract, references, figures and tables

For peer review only

ABSTRACT

Objectives Assess longitudinal associations between active travel during the school commute and later educational outcomes.

Setting England, Wales and Northern Ireland.

Participants 6778 children, surveyed at ages 7, 11, 14, and 17.

Primary and secondary outcomes School-leaver GCSE exam scores, summed to provide a single measure of educational success.

Results Controlling a range of sociodemographic and health variables, using active vs passive travel modes during a child's commute to school during earlier years predicted differences in school-leaver exam performance at age 16. These effects were mediated through changes in self-esteem, emotional difficulties and behavioural difficulties. Examples include: being driven to school at 11 was associated with improved exam performance at 16 mediated through enhanced self-esteem at 14 (*ab* = 0.08, 95% CI = 0.01 to 0.20, p = .05) and cycling at 14 was associated with better exam scores at 16 mediated through reduced emotional difficulty at 16 (*ab* = 0.10, 95% CI = 0.01 to 0.30, p = .05). The relationship between travel mode and exam performance was moderated by household income quintile, most notably with poorer exam performance seen in high-income children who were driven to school. Importantly, although our model predicted 21% of variance in exam performance, removing travel mode barely reduced its ability to predict exam scores (ΔR^2 = -.005, $F_{20,6469}$ = 2.50, p < .001).

Conclusion There are differences in school-leaver exam performance linked to travel mode choices earlier in the school career, but these differences are extremely small. There appears to

For peer review only - http://bmjopen.bmj.com/site/about/guidelines.xhtml

be no realistic educational disadvantage from any given travel mode, strengthening the case for cleaner, healthier modes to become the default.

Keywords: Environment and Public Health; Schools; Child Psychology; Travel; Cohort Analysis

For peer review only

For peer review only - http://bmjopen.bmj.com/site/about/guidelines.xhtml

ARTICLE SUMMARY

Strengths and limitations of this study

- Strength: We used the high-quality UK Millennium Cohort dataset, following a large sample of children over 10 years, enabling us to consider a broad range of health and demographic covariates.
- Strength: We used official, national-standard exam results as the outcome measure.
- Limitation: Data on travel to school were self-reported and reduced each child to a single 'main' mode, thereby not fully capturing the details of children whose journeys were more complex.
- Limitation: Data collection was at three-year intervals and so this dataset would not allow us to identify specific ages that are critical in development at anything other than a crude level.

For peer review only - http://bmjopen.bmj.com/site/about/guidelines.xhtml

INTRODUCTION

A child's daily journey to school provides routine opportunities for physical activity that might have important health consequences[1–3]. Active travel modes such as walking, cycling and scooting provide valuable daily exercise that is absent when children are driven to school, to say nothing of how driving children to school creates further serious public health problems through air-quality impairment. As well as obvious barriers to the adoption of more active travel for the school commute, such as geography and societal norms, there is a further barrier arising from the informal learning opportunities linked to the daily commute[4]. It has long been claimed that children who travel to school passively – like being chauffeured in cars – can miss out on everyday learning opportunities during their commutes compared to children who travel independently[4–6], and might also be less attentive upon arrival at school than children who use active modes like walking[3,7]. This might appear to make active travel the obvious choice except, in direct contrast to the studies just mentioned, it has also been claimed that being driven to school by adult caregivers every day provides children with important educational opportunities[6,8,9] from, for example, obtaining parental advice on homework. These conflicting claims, and a lack of evidence, mean policymakers cannot know the wider effects of supporting sustainable active travel to school (as is legally mandated in countries like the UK[10]). A strong test of this issue would be to ask whether any effects of travel to school can be picked up in official end-of-school exam grades, and this is what we provide here.

Although the mechanisms by which everyday travel might affect learning at school[4–6] have been discussed since the early 1990s[4–6], evidence in this area remains weak[11,12]. Five components of childhood wellbeing have been distinguished (physical, psychological, social, economic and cognitive[13]) – the last of which provides a plausible route between physical activity and school

For peer review only - http://bmjopen.bmj.com/site/about/guidelines.xhtml

performance[11]. A recent comprehensive review[12] suggested that cognitive ability, and thereby academic attainment, might be influenced by children's experiences on the journey to school through mechanisms like increased alertness[3] in walkers, cyclists and bus users, but noted that evidence remains anecdotal or inconsistent, a point also made recently elsewhere[11]. Enhanced peer relationships are another mechanism suggested to explain how independent travel might benefit children who walk or take buses to school[6,12], but there are also qualitative data from multiple sources suggesting that regular parental interaction for children who are escorted in cars could have educational benefits[6,8,9]. The contradictory claims in this area are impossible to resolve given the existing evidence[11,12] and so we performed a direct test of whether how children travel to school could predict their end-of-school exam scores several years later. The objective was to assess longitudinal associations between active travel during the school commute and later educational outcomes. Given the past research outlined here, we hypothesized that if we saw any educational advantages for users of certain travel modes, these might be separately mediated through changes in behavioural/attentional problems (which might plausibly be improved by regular physical activity[3,7,11,12]) and self-esteem (which might plausibly be improved from experiences of autonomy in school travel[4,6,7,11,12]).

METHODS

We tested our hypotheses using the Millennium Cohort Study dataset[14], a UK birth cohort study which tracks people from their birth in 2000-2001 to adulthood through multiple waves. The study used a cluster-stratified framework, oversampling groups such as those from the smaller nations of the UK, those from ethnic minority groups, and those from disadvantaged areas. The

For peer review only - http://bmjopen.bmj.com/site/about/guidelines.xhtml

entire dataset for the first wave comes from around 19,000 participants[14]. After dropouts, and after excluding cases in Scotland (where the exam system is different), cases with incomplete data on travel behaviour across ages 7, 11 and 14, and cases where travel mode was listed as 'other' or 'bike (someone else cycles)', we had records from 6778 participants who also provided data on GCSE (General Certificate of Secondary Education) or iGCSE (international GCSE) exams at age 16 (5033 from England, 1023 from Wales, 722 from Northern Ireland; 3078 male, 3423 female, 276 missing sex data). GCSEs provide a useful metric as they are intended to assess a broad range of the subjects learned at school and a set of (typically) 7-9 GCSE assessments has been undertaken by almost everybody in England, Wales and Northern Ireland at age 16 since the mid-1980s. We calculated a total numeric school-leaving exam score for each person using a government points system[15] to translate letter grades into numerical equivalents, giving greater credit to those who obtained higher grades and those who chose to take extra exams. The mean exam score for the sample was 46.27 points (SD = 21.87) which roughly corresponds to a child obtaining 5 B grades and 4 C grades[15].

The original dataset uses numerical codes to describe the following categories: public transport, school bus/coach, car/taxi, bike, bike (someone else cycles), walking, or refusal/don't know/not applicable/other. We reduced these to: "Public transport", "School bus or coach", "Private motorised", "Bike" and "Walk", and excluded the other categories from analysis. At age 7, travel to school was by the following modes: 3089 walking, 108 public transport, 198 school bus, 3329 private car and 54 bicycle. At age 14 the numbers were: 2462 walking, 1152 public transport, 1207 school bus, 1832 private car and 125 bicycle. Data on who the children travelled with on their way to school were only collected at age 11. However, there were clear signs that many respondents misunderstood this question and so the data were not used (of the 11-year-olds

For peer review only - http://bmjopen.bmj.com/site/about/guidelines.xhtml

travelling to school in cars, 2323 were recorded as travelling 'with adults' but 739 were 'with other children' and 150 'on their own'!).

We ran a series of causal mediation analyses[16] to assess plausible mechanistic pathways from travel mode to exam performance. We tested three potential mediators: Externalizing, which is a collective measure of inattentiveness, hyperactivity and conduct problems from the Strengths and Difficulties Questionnaire (SDQ)[17]; SDQ Internalizing, which measures emotional and peer-relationship difficulties[17]; and the Rosenberg Self-Esteem measure[18]. SDQ scores were parent-reported except at age 14 and 16, when they were child-reported. Each mediation analysis controlled for demographics and behavioural factors including sex, ethnicity, country of residence, distance from school, household income quintile, private schooling and – because physical activity was a plausible mechanism behind any travel-mode effects on educational success[3,8–10] – leisure-time sport and incidental physical activity. The Millennium Cohort contains weightings to enable descriptive statistics properly to represent the various countries within the UK, but these were not needed here given that we did not report simple descriptive statistics, and given country was included as a predictor in the analysis. The following temporal chains were assessed: travel at 7 → mediators at 11 → exams at 16; travel at 7 → mediators at 14 → exams at 16; travel at 11 → mediators at 14 → exams at 16; and travel at 14 → mediators at 16 → exams at 16. Mediation coefficients *ab* are interpreted as the mean marginal (i.e., controlling for covariates) change in exam score associated with the travel mode in question which is explained specifically through changes in the mediating variable[16]. Walking was set as the reference travel mode. Confidence intervals were estimated using nonparametric bootstrapping with 500 repetitions.

For peer review only - http://bmjopen.bmj.com/site/about/guidelines.xhtml

Linear regression analysis predicted total exam score for each child from travel behaviour at 7, 11 and 14 using the same demographic and behavioural covariates as the mediation analyses. The covariates were also tested for moderation effects with transport mode. A process of model simplification used backward deletion to remove statistically redundant predictors and interaction terms and so leave the minimal adequate model.

Patient and public involvement

Patients and the public were not involved in this study.

RESULTS

Our analysis found pathways between school travel mode and later exam performance, separately mediated through self-esteem, externalized behavior problems and internalized socio-emotional difficulties. The pathways with significant indirect mediated effects were as follows: cycling at 7 was associated with lower exam performance at 16 mediated through self-esteem changes at 11 (ab = -0.80, 95% CI = -1.61 to -0.12, p = .04); being driven at 11 was associated with improved exam performance at 16 mediated through enhanced self-esteem at 14 (ab = 0.08, 95% CI = 0.01 to 0.20, p = .05); being driven at 14 was associated with better exam performance at 16 mediated through enhanced self-esteem at 16 (ab = 0.19, 95% CI = 0.08 to 0.34, p < .001); public transport at 14 was associated with better exam scores at 16, also mediated through enhanced self-esteem at 16 (ab = 0.18, 95% CI = 0.04 to 0.36, p = .01); cycling at 14 was associated with worse exam scores at 16 mediated through greater externalizing at 16 (ab = -0.78, 95% CI = -1.54 to -0.06, p = .03), although cycling at 14 was also separately associated with better exam scores at 16 mediated through reduced internalizing at 16 (ab =

For peer review only - http://bmjopen.bmj.com/site/about/guidelines.xhtml

0.10, 95% CI = 0.01 to 0.28, p = .05). Reduced internalizing at 11 also mediated a relationship between public transport at 7 and increased exam scores at 16 (ab = 0.47, 95% CI = 0.02 to 0.96, p = .04). All other mediated pathways between travel history and exam scores were non-significant ($p \geq .10$).

A regression analysis (reported across Table 1 and Table 2) found that overall exam performance at 16 was related to the travel mode used to reach school two years earlier, and to demographic variables including sex and household income (R^2_{adj} = .21). Many of the control variable effects were expected (e.g., a mean marginal exam advantage of 6.30 points enjoyed by girls[19]), which supports the validity of the analysis. All minority ethnic groups performed substantially better than the reference white category and there were very clear cumulative advantages for children in higher-income households, peaking at a marginal mean difference of 24.46 points for the highest quintile versus the lowest (roughly equivalent to 8 exams each rising from grade C to grade A or to a child gaining three extra high-grade qualifications). The influence of travel mode was significantly moderated by household income quintile (Fig. 1); perhaps the most notable aspect of this interaction was that, in the highest household income group, there was a mean marginal reduction of 4.63 exam points (equivalent to three exams each dropping from an A to a B) for the children who were driven to school (Fig. 1).

Critically, however, entirely removing travel mode from our model barely changed its overall ability to predict exam scores (ΔR^2 = -.005, $F_{20,6469}$ = 2.50, $p < .001$). This reveals travel mode to be a trivial influence on exam scores compared to the demographic and behavioural covariates. This accords with the small effect sizes described above, e.g., being driven at 14 led to a mean mediated change in exam scores of just 0.20 points across all exams versus walking, which is extremely small against an overall mean exam score of 46.27 points.

For peer review only - http://bmjopen.bmj.com/site/about/guidelines.xhtml

DISCUSSION

This analysis has shown for the first time that there are specific psycho-behavioural pathways linking travel to school in teenage years with later academic outcomes, as assessed through end-of-school exam results. The pathways were particularly mediated through changes in self-esteem, and to some extent behavioral and socio-emotional difficulties, that in turn were influenced by travel experiences earlier in a child's school career. Prior work has distinguished two domains that might be associated with daily travel: psychological (wellbeing, independence) and cognitive (mental ability, academic attainment)[11,12]. The present study has demonstrated that these two domains interact such that the psychological domain might have an effect on the cognitive domain. It is notable that being driven to school by adult caregivers at 14 and independently using public transport at 14 both produced similar effects on later exam performance through a route involving enhanced self-esteem. This implies that, contrary to past claims[4–6,8,9], it cannot simply be quality time with adults *or* independent mobility that uniquely boosts school success. Rather, there appears to be more than one route to better school outcomes as a result of daily travel experiences and both active and passive modes can provide some of these benefits provided they have immediate effects on mediating psychological factors like self-esteem, emotional adjustment or behavioural difficulties.

That last point notwithstanding, this study's core finding is to show, using an adequately powered sample, that while components of childhood wellbeing like self-esteem and socio-emotional difficulties[11–13] can be influenced by the way a child travels to school, and that while these in turn might influence exam success, the magnitude of this effect on school outcomes is

For peer review only - http://bmjopen.bmj.com/site/about/guidelines.xhtml

dramatically smaller than the influences of demographics, health and socio-economic status (cf.[12]). Once these covariates were controlled, the way children travelled to school explained just 0.5% of unique variance in exam outcomes. Given the size and quality of the sample used here, we can state with some confidence that the way children travel to school is a real, but very minor, influence on their scholastic success.

This study draws strength from its large, representative, high-quality longitudinal sample and its use of an official, national-standard set of examinations – taken by practically everybody in the three countries studied – as the outcome measure. Limitations attach to the self-report measures of travel mode (in particular we identified concerns with the quality of some of the data on adult escorts) and our method needing to reduce each child to a single mode, thereby likely missing nuances of children who have more complex school journeys, such as returning home using a different mode.

CONCLUSION

Our finding that there is no clear educational advantage for any one travel mode should end some long-standing speculation within the research literature about the impact of travel experiences on learning[3–9,11–13] and thereby help unlock public health gains by removing an impediment to reducing the use of private cars for the school commute. Modes like walking, cycling and buses are immediately positive for children's wellbeing[20] and also provide health benefits[1–2], so it is perhaps surprising they do not translate into more substantial academic performance advantages. On the other hand, as we have clearly shown here, nor does spending time in cars with adult caregivers. Given the known negative consequences of driving children to

For peer review only - http://bmjopen.bmj.com/site/about/guidelines.xhtml

school, including air pollution and lack of physical activity[1,2,11], it is now clear that policymakers can recommend active and public transport modes as the default choice without fear of meaningful long-term educational disadvantage.

Acknowledgments We thank Anthony Laverty for his advice on working with the Millennium Cohort dataset.

Author Contributions Conceptualization: IW, TG; Methodology: IW, TG; Data management: TG; Statistical analysis: IW; Visualization: IW; Writing – original draft: IW; Writing – review & editing: IW, TG

Funding This study/project did not receive external funding.

Disclaimer The views expressed are those of the author(s) and not necessarily those of Swansea University or the University of Surrey.

Competing interests None declared.

Patient consent for publication Not required.

Ethics approval As this study involved secondary analyses of data that do not contain identifiable information, the MCS ethical approval was not required. Data collection for the MCS has ethical approval from the Yorkshire and Humber ethics committee (11/YH/0203) and further details are available from http://www.cls.ioe. ac.uk/. Participants give consent for their data to be used for research purposes.

Provenance and peer review Not commissioned; externally peer reviewed.

For peer review only

For peer review only - http://bmjopen.bmj.com/site/about/guidelines.xhtml

Data availability statement MCS data are available from https://ukdataservice.ac.uk/. Study pre-registration available from https://osf.io/u9wdr. Analysis code available from https://osf.io/46eq3.

Supplemental material This content has been supplied by the author(s). It has not been vetted by BMJ Publishing Group Limited (BMJ) and may not have been peer-reviewed. Any opinions or recommendations discussed are solely those of the author(s) and are not endorsed by BMJ. BMJ disclaims all liability and responsibility arising from any reliance placed on the content. Where the content includes any translated material, BMJ does not warrant the accuracy and reliability of the translations (including but not limited to local regulations, clinical guidelines, terminology, drug names and drug dosages), and is not responsible for any error and/or omissions arising from translation and adaptation or otherwise.

Open access This is an open access article distributed in accordance with the Creative Commons Attribution 4.0 Unported (CC BY 4.0) license, which permits others to copy, redistribute, remix, transform and build upon this work for any purpose, provided the original work is properly cited, a link to the licence is given, and indication of whether changes were made. See: https://creativecommons.org/ licenses/by/4.0/.

ORCID iDs

Ian Walker https://orcid.org/0000-0002-0079-3149

Tim Gamble https://orcid.org/0000-0002-6610-0835

REFERENCES

For peer review only - http://bmjopen.bmj.com/site/about/guidelines.xhtml

1 Tudor-Locke C, Ainsworth BE, Popkin BM. Active commuting to school: an overlooked source of children's physical activity? *Sports Med* 2001;31:309–313.

2 Laverty AA, Hone T, Goodman A, Kelly Y, Millett C. Associations of active travel with adiposity among children and socioeconomic differentials: a longitudinal study. *BMJ Open* 2021;11:e036041.

3 Mackett RL, Lucas L, Paskins J, Turbin J. The therapeutic value of children's everyday travel. *Trans Res Part A* 2005;39:205–219.

4 Kegerreis S. Independent mobility and children's mental and emotional development. In Hillman M, ed. Children, Transport and the Quality of Life. London: *Policy Studies Institute*, 1993:28–24.

5 Hillman M, Adams J, Whitelegg J. One False Move: A Study of Children's Independent Mobility. London: *Policy Studies Institute*, 1990.

6 Gough D, Oliver D, Brunton G, Selai C, Schaumberg H. The effect of travel modes on children's mental health, cognitive and social development: A systematic review. London: *Evidence for Policy and Practice Information and Co-ordinating Centre*, 2001.

7 Cooper AR, Andersen LB, Wedderkopp N, Page AS, Froberg K. Physical activity levels of children who walk, cycle or are driven to school. *Am J Prev Med* 2005;29:179–184.

8 Barker J. 'Manic mums' and 'distant dads'? Gendered geographies of care and the journey to school. *Health & Place* 2011;17:413–421.

For peer review only

For peer review only - http://bmjopen.bmj.com/site/about/guidelines.xhtml

9 Yeung R, Nguyen-Hoang P. It's the journey, not the destination: The effect of school travel mode on student achievement. *J Urbanism: Int Res on Placemaking and Urban Sustainability* 2020;13:170–186.

10 UK Government. Section 508A, Education Act, 1996. Available: https://www.legislation.gov.uk/ukpga/1996/56/section/508A

11 Westman J, Friman M, Olsson LE. Travel and child wellbeing: The psychological and cognitive domains. In Owen E, Waygood D, Friman M, Olsson LE, Mitra R, eds. Transport and Children's Wellbeing. *Elsevier*, 2020:41–59.

12 Waygood EOD, Friman M, Olsson LE, Taniguchi A. Transport and child well-being: An integrative review. *Trav Beh and Soc* 2017;9:32–49.

13 Pollard E, Lee P. Child well-being: A systematic review of the literature. *Social Indicators Research* 2003;61:59–78.

14 Joshi H., Fitzsimons E. The Millennium Cohort Study: The making of a multi-purpose resource for social science and policy. *Longitud. Life Course Stud.* 2016;7:409-430.

15 UK Department for Education. GCSE factsheet for parents, 2019. Available: https://www.gov.uk/government/publications/gcse-new-grading-scale-factsheets

16 Hayes AF, Preacher KJ. Statistical mediation analysis with a multicategorical independent variable. *Brit J Math Stat Psych* 2014;67:451–470.

17 Goodman R. The strengths and difficulties questionnaire: A research note. *J Child Psych Psychiat* 1997;38:581–586.

For peer review only

For peer review only - http://bmjopen.bmj.com/site/about/guidelines.xhtml

18 Rosenberg M. Rosenberg Self-Esteem Scale. *APA PsycTests*, 1965.

19 Angelo C, Reis AB. Gender gaps in different grading systems. *Edu Econ* 2021;29:105–119.

20 Westman J, Olsson LE, Gärling T, Friman M. Children’s travel to school: satisfaction, current mood, and cognitive performance. *Transportation* 2017;44:1365–1382.

For peer review only

For peer review only - http://bmjopen.bmj.com/site/about/guidelines.xhtml

Table 1. Linear regression model predicting total school exam scores at age 16 from demographic and travel variables. Part 1: Demographics

		Coeff.	Std err.	*t*	*p*	95% CI lower	95% CI upper
	(Intercept)	26.46	1.39	19.03	<.001	23.74	29.19
Country	England	ref					
	Wales	4.25	0.72	5.92	<.001	2.84	5.66
	Northern Ireland	3.71	0.84	4.41	<.001	2.06	5.36
Sex	Male	ref					
	Female	6.31	0.49	12.80	<.001	5.35	7.28
Ethnicity	White	ref					
	Mixed	3.44	1.46	2.35	.02	0.57	6.31
	Indian	8.93	1.41	6.31	<.001	6.15	11.70
	Pakistani/Bangladeshi	9.49	1.16	8.18	<.001	7.21	11.77
	Black	4.78	1.51	3.16	.002	1.82	7.75
	Other inc. Chinese	16.63	1.93	8.64	<.001	12.86	20.41
Household income quintile	1 (lowest)	ref					
	2	5.09	1.51	3.37	<.001	2.13	8.06
	3	10.35	1.40	7.41	<.001	7.61	13.09

For peer review only - http://bmjopen.bmj.com/site/about/guidelines.xhtml

	4	15.01	1.37	10.98	<.001	12.33	17.69
	5 (highest)	24.47	1.38	17.74	<.001	21.77	27.18
Mental health issue	No	ref					
	Yes	-4.39	1.10	3.99	<.001	-6.55	-2.23
Longstanding illness	No	ref					
	Yes	-2.83	0.73	3.88	<.001	-4.25	-1.40
Private schooling	No	ref					
	Yes	2.77	0.93	2.98	.003	0.95	4.58
Sport at age 7	per 1 point increment	1.60	0.21	7.66	<.001	1.19	2.01
Sport at age 11	per 1 point increment	0.99	0.17	5.77	<.001	0.65	1.33
Other physical activity at 7	per 1 point increment	-0.40	0.15	2.67	.008	-0.70	-0.11
Other physical activity at 11	per 1 point increment	-0.50	0.14	3.50	<.001	-0.79	-0.22

Note: The travel components of this model are found in Table 2. Statistically redundant predictors and interaction terms have been removed.

For peer review only - http://bmjopen.bmj.com/site/about/guidelines.xhtml

Table 2. Linear regression model predicting total school exam scores at age 16 from demographic and travel variables. Part 2: Travel behaviour

		Coeff.	Std err.	*t*	*p*	95% CI lower	95% CI upper
Transport mode age 14	Walking	ref					
	Public transport	2.04	1.92	1.06	.29	-1.73	5.81
	School bus or coach	-2.54	2.58	0.99	.32	-7.59	2.51
	Private motorised	0.17	1.67	0.10	.92	-3.10	3.45
	Cycling	9.12	6.24	1.46	.14	-3.12	21.35
Age 14 public transport (v. walking) x household income quintile 2 (v. 1)		-1.22	2.73	0.45	.65	-6.58	4.13
Age 14 public transport (v. walking) x household income quintile 3 (v. 1)		-0.98	2.47	0.40	.69	-5.82	3.86
Age 14 public transport (v. walking) x household income quintile 4 (v. 1)		1.11	2.41	0.46	.64	-3.60	5.83
Age 14 public transport (v. walking) x household income quintile 5 (v. 1)		-2.88	2.34	1.23	.22	-7.46	1.70
Age 14 school bus (v. walking) x household income quintile 2 (v. 1)		5.95	3.21	1.85	.06	-0.34	12.24
Age 14 school bus (v. walking) x household income quintile 3 (v. 1)		4.66	2.99	1.56	.12	-1.20	10.52

For peer review only

Age 14 school bus (v. walking) x household income quintile 4 (v. 1)	6.50	2.91	2.24	.03	0.81	12.20
Age 14 school bus (v. walking) x household income quintile 5 (v. 1)	1.99	2.87	0.69	.49	-3.64	7.61
Age 14 private motorised (v. walking) x household income quintile 2 (v. 1)	1.54	2.42	0.63	.53	-3.21	6.28
Age 14 private motorised (v. walking) x household income quintile 3 (v. 1)	0.38	2.17	0.18	.86	-3.87	4.63
Age 14 private motorised (v. walking) x household income quintile 4 (v. 1)	1.61	2.07	0.78	.44	-2.45	5.67
Age 14 private motorised (v. walking) x household income quintile 5 (v. 1)	-4.64	2.03	2.28	.02	-8.62	-0.67
Age 14 cycling (v. walking) x household income quintile 2 (v. 1)	-16.33	8.45	1.93	.05	-32.90	0.23
Age 14 cycling (v. walking) x household income quintile 3 (v. 1)	-19.70	7.49	2.63	.009	-34.38	-5.02
Age 14 cycling (v. walking) x household income quintile 4 (v. 1)	-2.22	7.24	0.31	.76	-16.43	11.97
Age 14 cycling (v. walking) x household income quintile 5 (v. 1)	-12.85	6.91	1.86	.06	-26.39	0.69

Note: The demographic components of this model are found in Table 1. Statistically redundant predictors and interaction terms have been removed.

For peer review only - http://bmjopen.bmj.com/site/about/guidelines.xhtml

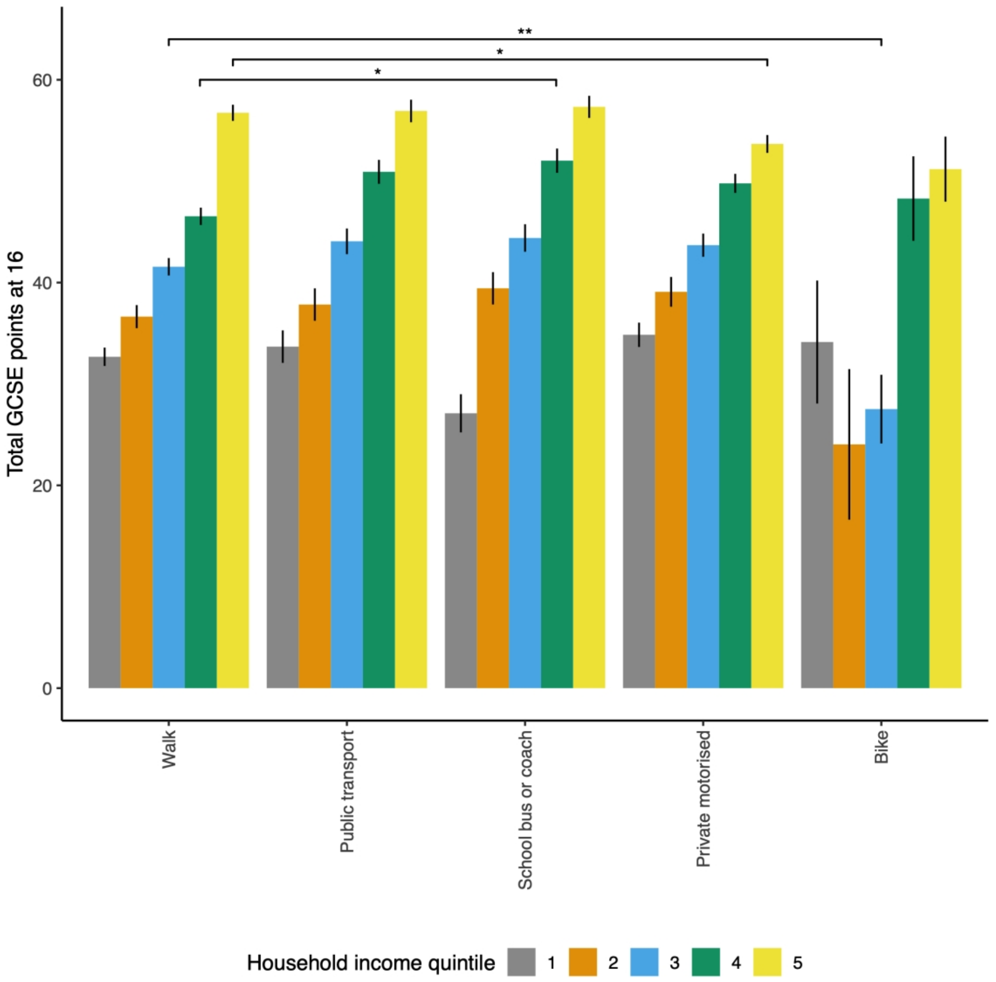

Fig. 1 School exam scores at age 16 from N = 6778 children according to household income quintile and travel mode at age 14. Walking was set as the travel reference category. * $p < .05$, ** $p < .01$

199x199mm (300 x 300 DPI)

STROBE 2007 (v4) Statement—Checklist of items that should be included in reports of *cohort studies*

Section/Topic	Item #	Recommendation	Reported on page #
**Title and abstract**	1	(*a*) Indicate the study's design with a commonly used term in the title or the abstract	1-2
		(*b*) Provide in the abstract an informative and balanced summary of what was done and what was found	2
**Introduction**			
Background/rationale	2	Explain the scientific background and rationale for the investigation being reported	5-6
Objectives	3	State specific objectives, including any prespecified hypotheses	2, 6
**Methods**			
Study design	4	Present key elements of study design early in the paper	6-7
Setting	5	Describe the setting, locations, and relevant dates, including periods of recruitment, exposure, follow-up, and data collection	6-7
Participants	6	(*a*) Give the eligibility criteria, and the sources and methods of selection of participants. Describe methods of follow-up	7
		(*b*) For matched studies, give matching criteria and number of exposed and unexposed	na
Variables	7	Clearly define all outcomes, exposures, predictors, potential confounders, and effect modifiers. Give diagnostic criteria, if applicable	7-8
Data sources/ measurement	8*	For each variable of interest, give sources of data and details of methods of assessment (measurement). Describe comparability of assessment methods if there is more than one group	6-8
Bias	9	Describe any efforts to address potential sources of bias	na
Study size	10	Explain how the study size was arrived at	6-7
Quantitative variables	11	Explain how quantitative variables were handled in the analyses. If applicable, describe which groupings were chosen and why	6-8
Statistical methods	12	(*a*) Describe all statistical methods, including those used to control for confounding	8-9
		(*b*) Describe any methods used to examine subgroups and interactions	8-9
		(*c*) Explain how missing data were addressed	6-7
		(*d*) If applicable, explain how loss to follow-up was addressed	na
		(*e*) Describe any sensitivity analyses	na

For peer review only

**Results**			
Participants	13*	(a) Report numbers of individuals at each stage of study—eg numbers potentially eligible, examined for eligibility, confirmed eligible, included in the study, completing follow-up, and analysed	6-7
		(b) Give reasons for non-participation at each stage	6-7
		(c) Consider use of a flow diagram	na
Descriptive data	14*	(a) Give characteristics of study participants (eg demographic, clinical, social) and information on exposures and potential confounders	6-8
		(b) Indicate number of participants with missing data for each variable of interest	6-7
		(c) Summarise follow-up time (eg, average and total amount)	6-7
Outcome data	15*	Report numbers of outcome events or summary measures over time	6-7
Main results	16	(*a*) Give unadjusted estimates and, if applicable, confounder-adjusted estimates and their precision (eg, 95% confidence interval). Make clear which confounders were adjusted for and why they were included	18-21
		(*b*) Report category boundaries when continuous variables were categorized	na
		(*c*) If relevant, consider translating estimates of relative risk into absolute risk for a meaningful time period	na
Other analyses	17	Report other analyses done—eg analyses of subgroups and interactions, and sensitivity analyses	na
**Discussion**			
Key results	18	Summarise key results with reference to study objectives	11
**Limitations**			
Interpretation	20	Give a cautious overall interpretation of results considering objectives, limitations, multiplicity of analyses, results from similar studies, and other relevant evidence	11-12
Generalisability	21	Discuss the generalisability (external validity) of the study results	11-12
**Other information**			
Funding	22	Give the source of funding and the role of the funders for the present study and, if applicable, for the original study on which the present article is based	13

*Give information separately for cases and controls in case-control studies and, if applicable, for exposed and unexposed groups in cohort and cross-sectional studies.

Note: An Explanation and Elaboration article discusses each checklist item and gives methodological background and published examples of transparent reporting. The STROBE checklist is best used in conjunction with this article (freely available on the Web sites of PLoS Medicine at http://www.plosmedicine.org/, Annals of Internal Medicine at http://www.annals.org/, and Epidemiology at http://www.epidem.com/). Information on the STROBE Initiative is available at www.strobe-statement.org.

For peer review only
